# Mechanical Properties and Crack Evolution of Rock-like Materials with Varying Particle Sizes Prepared by Impact and Static Compaction Methods

**DOI:** 10.3390/ma18081695

**Published:** 2025-04-08

**Authors:** Qi Hu, Qinglin Li, Xingyan Liu, Kun Ding, Zongen Li, Yuxiang Bai

**Affiliations:** 1 College of Water Conservancy & Architectural Engineering, Shihezi University, Shihezi 832003, China; hooo7777@163.com (Q.H.); liql1150142@163.com (Q.L.); lizongen2022@163.com (Z.L.); baiyuxiang@xjshzu.com (Y.B.); 2 Key Laboratory of Cold and Arid Regions Eco-Hydraulic Engineering of Xinjiang Production & Construction Corps, Shihezi University, Shihezi 832003, China; 3 China Construction Eighth Engineering Bureau Southwest Company, Chengdu 610041, China; dingkun@tju.edu.cn

**Keywords:** static compaction method, impact compaction method, DIC technique, crack propagation, microstructural characteristics

## Abstract

To investigate the effects of impact and static compaction methods on the mechanical properties and crack evolution of rock-like materials with varying particle sizes. Uniaxial compression tests combined with Digital Image Correlation (DIC) technology were conducted on specimens of two aeolian sand gradations (0.075–0.18 mm and 0.22–0.5 mm) and one quartz sand gradation (0.22–0.5 mm). The study focused on elastic modulus, peak strength, stress-strain behavior, failure modes, surface deformation fields, crack propagation paths, and strain evolution at characteristic points under both compaction methods. Finally, the microstructure of specimens was analyzed and compared with natural rock analogs. Key results include: (1) At an identical density of 1.82 g/cm^3^, static-compacted specimens of fine-grained aeolian sand (0.075–0.18 mm) exhibited higher elastic modulus and peak strength compared to impact-compacted counterparts, whereas inverse trends were observed for coarse-grained aeolian sand (0.22–0.5 mm) and quartz sand specimens; (2) Under equivalent compaction energy (254.8 J), the hierarchy of mechanical performance was: quartz sand > coarse-grained aeolian sand > fine-grained aeolian sand; (3) Static-compacted specimens predominantly failed through tensile splitting, while impact-compacted specimens exhibited shear-dominated failure modes; (4) DIC full-field strain mapping revealed rapid propagation of primary cracks along pre-existing weak planes in static-compacted specimens, forming through-going tensile fractures. In contrast, impact-compacted specimens developed fractal strain field structures with coordinated evolution of shear bands and secondary tensile cracks; (5) Microstructural comparisons showed that static-compacted fine-grained aeolian sand specimens exhibited root-like structures with high porosity, resembling weakly consolidated sedimentary rocks. Impact-compacted coarse-grained aeolian sand specimens displayed stepped structures with dense packing, analogous to strongly cemented sandstones.

## 1. Introduction

Rocks are composite aggregates composed of minerals with varying types and particle sizes arranged in specific configurations. Their mechanical properties are governed by mineral composition, particle size distribution, and specimen preparation methods, with strength and deformation-failure characteristics remaining central research priorities in rock mechanics [1,2,3,4,5,6,7,8,9,10,11]. However, significant controversies persist in the preparation of artificial rock-like materials. Although the Standard for Geotechnical Testing Methods (GB/T 50123-2019) [12] recommends impact compaction (dynamic) and static compaction (quasi-static) for fabricating cylindrical specimens, systematic investigations into the differences in forming mechanisms between materials of varying particle sizes (e.g., aeolian sand vs. quartz sand) and their systemic impacts on elastic modulus, peak strength, stress-strain behavior, failure modes, surface deformation fields, crack propagation paths, and strain evolution at characteristic points remain scarce [13,14,15]. Existing studies predominantly focus on single-size aggregates, partial particle substitutions, or singular preparation methods, failing to elucidate the triadic relationships among particle size, preparation methodology, and failure characteristics, thereby limiting the fidelity of synthetic rock analogs. This study addresses these gaps by comparing the mechanical properties of aeolian sand and quartz sand with two particle size ranges—0.075–0.18 mm (fine-grained) and 0.22–0.5 mm (coarse-grained)—under both compaction methods. Quantitative characterization of failure modes and full-field strain analysis via Digital Image Correlation (DIC) were performed to evaluate methodology-dependent behaviors. A key limitation lies in the exclusive use of standard specimen dimensions (D = 50 mm, H = 100 mm); large-scale specimens will be prioritized in subsequent research phases.

Researchers have extensively utilized the impact compaction method in uniaxial and triaxial experimental investigations: Qingsong Zheng et al. [16] conducted triaxial compression tests on jointed rock specimens with varying structural plane inclinations, analyzing their effects on mechanical properties, volumetric contraction, and dilation behaviors. Mingxing Liu et al. [17] performed single-frequency cyclic triaxial tests on artificial jointed specimens, investigating the mechanical response under different joint dip angles. Qingsong Zheng’s [18] team further explored frequency-dependent mechanical degradation through multi-frequency cyclic triaxial loading tests on specimens with artificial joints. Mingxing Liu et al. [19] employed impact-compacted specimens to reveal damage evolution mechanisms in jointed rock masses under cyclic loading. Cheng Pu et al. [20] developed vibration-compacted specimens with non-persistent fractures, studying the influence of confining pressure and fracture dip angles on mechanical performance. The static compaction method has been widely applied to soil and frozen soil specimen preparation: Xiangchuan Meng et al. [21] fabricated remolded soil specimens using static compaction in cutting rings for low-temperature cyclic freezing-thawing experiments. Xingyan Liu et al. [22] produced frozen composite soils via static compaction, analyzing the impact of coarse particle content on mechanical properties. Xiangtian Xu et al. [23] conducted triaxial compression tests on statically compacted saline frozen silty sand (−6 °C, 0.5% salinity) under confining pressures ranging from 0.3 to 18 MPa. For synthetic rock interfaces, Yanbo Zhu et al. [24] prepared siliceous slate-mudstone rough contact specimens by compacting remolded mudstone against oriented slate surfaces, demonstrating the method’s adaptability for simulating complex lithological interfaces.

The Impact of Particle Size on Rock-like Materials: Shuangxi Yuan et al. [25] conducted compression-shear tests on square rock-like specimens with different pre-existing fracture patterns and varying particle size compositions. Their research revealed the influence of different particle size components on the peak shear stress of rock-like materials and the corresponding shear displacement. Gang Wang et al. [26] carried out direct shear experiments to explore the relationship between localized stress and granular behavior, clarifying the impact of particle size on the mechanical properties of granular-filled joints. Zhe Zhang et al. [27] proposed a method to improve the mechanical properties of SP-3DP (sandstone-like 3D printed) rock-like specimens by optimizing particle gradation. Through a series of mechanical tests, acoustic emission analysis, and scanning electron microscope observations, the impact of different particle sizes and gradations on the mechanical properties of the specimens was investigated. Hao Qin et al. [28] studied the effect of particle size distribution on the flexural performance and microstructural characteristics of fiber-reinforced cemented tailings backfill (FRCTRF). H. Beshr et al. [29] conducted a study to evaluate the effect of four types of coarse aggregates—calcareous limestone; dolomitic limestone; quartzitic limestone; and steel slag—on the compressive strength; tensile strength; and elastic modulus of high-strength concrete. Among these, the concrete specimens prepared with steel slag aggregates exhibited the highest compressive strength, while those with calcareous limestone aggregates showed the lowest compressive strength. Nguyen, L.H. et al. [30] used seven types of fine and coarse lightweight aggregates from three different sources. The aggregates in mixes were a combination of coarse lightweight aggregate with fine normal-weight aggregate and/or fine lightweight aggregate. The study indicated that the replacement of fine normal-weight aggregate by fine lightweight aggregate reduces concrete strength but improves its thermal performance. Kui Zhao et al. [31] prepared rock-like material specimens with four different particle sizes to investigate the influence of particle size composition on the acoustic emission (AE) characteristics of materials subjected to tensile failure under three-point bending. The mechanical properties and AE characteristics of each specimen during loading were analyzed.

Previous studies on the impact and static compaction methods have primarily focused on investigating the influence of individual factors—such as joint inclination; multi-fracture networks; confining pressure; and cyclic loading—on the mechanical properties of rock-like specimens under a single preparation method. However, comparative research on the mechanical differences between specimens prepared by these two methods remains limited, particularly regarding the effects of material composition and particle size on the mechanical performance of artificial rocks. A deeper understanding of the mechanical disparities induced by different preparation methods is crucial for rationally selecting methodologies to fabricate idealized rock analogs. To address this gap, further research is necessary to systematically explore the effects of preparation methods and particle size on the mechanical properties and crack evolution of specimens through uniaxial compression tests combined with Digital Image Correlation (DIC) and Scanning Electron Microscopy (SEM) techniques. Comparative analysis will clarify the applicability of impact and static compaction methods for simulating specific rock types (e.g., high-porosity low-strength rocks vs. dense high-strength lithologies) while providing novel insights for refining rock mechanics testing standards.

## 2. Materials and Methods

### 2.1. Materials and Specimen Preparation

The experimental materials comprised aeolian sand, quartz sand, Portland cement, and barium sulfate powder. Aeolian sand refers to sand layers transported and deposited by wind action, commonly found in desert and gobi regions. The aeolian sand, sourced from the Ehen Hudag Railway traversing desert-gobi terrain, exhibited primary chemical constituents including SiO_2_, Al_2_O_3_, CaO, and Fe_2_O_3_, with detailed physical properties documented in Reference [32], as shown in Table 1. The quartz sand was sourced from a high-purity quartz sand plant in Zhengzhou City, Henan Province. The primary chemical composition of the quartz sand is listed in Table 2, with a bulk density of 1.73 g·cm^−3^ and a specific gravity of 2.66 g·cm^−3^. A P·O 52.5 high-grade cement with a specific surface area of 376 m^2^/kg served as the cementitious binder.

**Figure 1:**
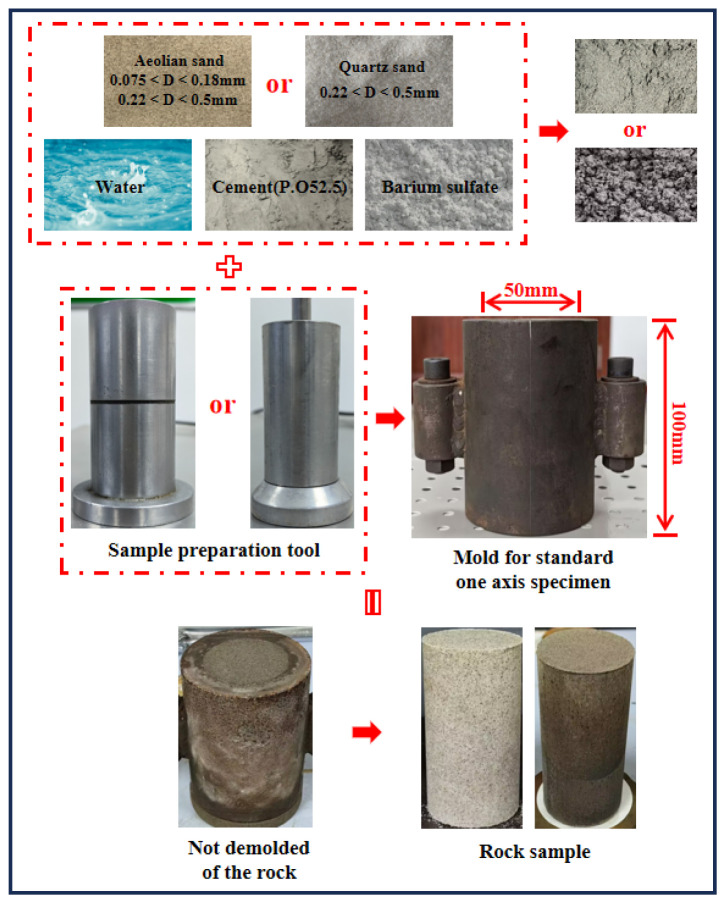
Preparation mold and specimen.

The specimen mold and prepared specimens are illustrated in Figure 1. The cylindrical mold has a height of 100 mm and an internal diameter of 50 mm. Prior to testing, aggregates were sieved into specific size ranges: aeolian sand was divided into two fractions (0.075–0.18 mm and 0.22–0.5 mm), while quartz sand was classified within the 0.22–0.5 mm range. The material mixture, designated as Mixture X, consisted of cement, water, aggregate, and barium sulfate powder uniformly blended at a mass ratio of 5:2:15:1.5.

For the static compaction method, Mixture X was poured into the mold, which was equipped with a forming head at its base. Specimen consolidation was achieved through the coordinated action of a hydraulic jack and the forming head. In the impact compaction method, Mixture X was layered into the mold in four equal increments, with each layer compacted to a precise height of 2.5 mm based on preliminary optimization trials.

Following molding, specimens were transferred to a standard curing chamber (temperature: 20 ± 2 °C, relative humidity: 95%). Demolding was conducted after 9 h of initial curing, after which specimens were returned to the curing chamber. Mechanical testing commenced after a total curing period of 3 days.

### 2.2. Experimental Procedure and Apparatus

**Figure 2:**
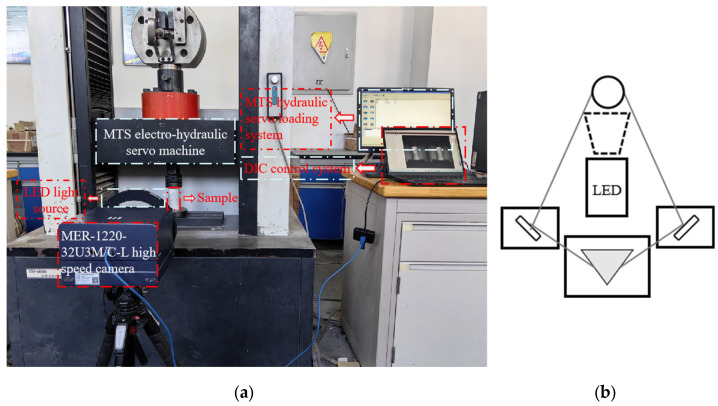
Test system and its schematic diagram for three-dimensional strain monitoring. (**a**) Sample and test system; (**b**) Three-dimensional strain detection optical path system.

The experimental apparatus, as depicted in Figure 2a, consists of a loading system and a digital image correlation (DIC) acquisition system. DIC, a non-contact optical measurement technique, captures full-field displacement and strain distributions by tracking speckle pattern displacements within a predefined region of interest (ROI) using high-speed cameras [33]. The loading system employed a CSS-44300 computer-controlled electro-hydraulic servo universal testing machine, operating under displacement control at a rate of 1.2 mm/min until complete specimen failure. The DIC system integrated MER-1220-32U3M/C-L high-speed cameras (resolution: 4024 × 3036 pixels; sensor: 1/1.7″ Sony IMX226 CMOS with rolling shutter), image acquisition software, and high-intensity LED illumination, achieving a frame rate of 32.3 fps. All DIC-generated images presented in the manuscript were produced under the three-dimensional mapping mode. Prior to testing, specimen surfaces were prepared by cleaning, applying a uniform white matte base coat, and spraying a high-contrast black speckle pattern to meet DIC measurement requirements. This configuration ensured synchronized mechanical loading and strain field analysis, enabling precise characterization of deformation and fracture dynamics. Figure 2b illustrates the optical system for three-dimensional strain detection, comprising a monocular three-dimensional image acquisition system and a three-dimensional strain detection system. Key components include a light source, triangular prism, reflector, and others. This system simplifies the structural complexity of three-dimensional strain detection setups while enhancing detection efficiency.

We conducted uniaxial compression tests on specimens prepared using two aeolian sand gradations (0.075–0.18 mm and 0.22–0.5 mm) and one quartz sand gradation (0.22–0.5 mm) under both static and impact compaction methods. Four comparative analyses were systematically designed: (1) Compaction Method Comparison: Identical-density specimens prepared via static vs. impact compaction were compared to evaluate method-dependent mechanical responses (key parameters in Table 3). (2) Static Compaction Material Effects: Static-compacted specimens with matched densities but differing aggregate types/sizes were analyzed (Table 4). (3) Impact Compaction Material Effects: Impact-compacted specimens with identical densities but varying aggregates were contrasted (Table 5). (4) Compaction Energy Equivalence: Specimens compacted with equivalent energy (50 strikes per layer) were compared to isolate particle-size influences (Table 6). To mitigate data scatter and enhance the reliability of experimental results, three replicate specimens were fabricated and tested for each type of rock-like material.

## 3. Mechanical and Failure Characteristics Analysis

### 3.1. Elastic Modulus and Strength Characteristics

The elastic modulus, a critical parameter reflecting a rock’s deformation resistance under load, serves as a fundamental indicator for evaluating mechanical performance and stability. Key findings from the experimental data are summarized as follows: (1) From Table 3, the following conclusions can be drawn: Under identical aggregate composition and particle size conditions, the static compaction method yields specimens with a higher elastic modulus for fine-grained aeolian sand (0.075–0.18 mm) compared to the impact compaction method. Conversely, for specimens prepared with coarse-grained aeolian sand (0.22–0.5 mm) and quartz sand, the elastic modulus demonstrated an inverse trend, with the impact compaction method producing higher values. (2) Static Compaction Effects (Table 4): Coarse-grained aeolian sand specimens under static compaction demonstrated an elastic modulus of 1.963 GPa, representing reductions of 9.8% and 11.1% compared to fine-grained aeolian sand and quartz sand specimens, respectively. (3) Impact Compaction Effects (Table 5): Coarse-grained aeolian sand specimens prepared via impact compaction exhibited an elastic modulus of 2.473 GPa, 13.7% lower than quartz sand counterparts. Notably, impact-compacted coarse-grained aeolian sand showed a 71.7% modulus enhancement relative to its fine-grained counterpart, contrasting sharply with static compaction trends. (4) Energy-Equivalent Compaction (Table 6): Under identical compaction energy, elastic modulus values for coarse-grained aeolian sand and quartz sand specimens differed by less than 1%, aligning with trends observed in Table 5 but demonstrating reduced material-specific divergence. (5) From Table 4, Table 5 and Table 6, it is evident that specimens fabricated with quartz sand consistently exhibited higher elastic modulus values compared to those prepared with aeolian sand across all three specimen types.

In rock mechanics, the initial yield strength marks the critical transition from elastic to plastic deformation under loading, while the peak strength represents the maximum compressive stress a specimen can withstand. As demonstrated in Table 3, Table 4, Table 5 and Table 6, the trends governing initial yield strength and peak strength are consistent across all tested conditions. For conciseness, the strength laws are summarized below using peak strength as the representative parameter: (1) From Table 3, the following conclusions can be drawn: For identical aggregate types and particle sizes, the peak strength of specimens prepared with fine-grained aeolian sand (0.075–0.18 mm) using the static compaction method exceeds that of those fabricated via the impact compaction method. Conversely, specimens prepared with coarse-grained aeolian sand (0.22–0.5 mm) and quartz sand exhibit the opposite trend. (2) Static Compaction Effects (Table 4): Coarse-grained aeolian sand specimens under static compaction showed a peak strength of 6.57 MPa, representing reductions of 27.6% and 33.8% compared to fine-grained aeolian sand and quartz sand specimens, respectively. (3) Impact Compaction Effects (Table 5): Coarse-grained aeolian sand specimens achieved a peak strength of 7.7 MPa, marginally exceeding fine-grained counterparts by 3%. Quartz sand specimens outperformed coarse-grained aeolian sand by 46.5% in peak strength. (4) Energy-Equivalent Compaction (Table 6): Under matched compaction energy, peak strength trends aligned with Table 5, though differences between coarse-grained aeolian sand and quartz sand specimens diminished significantly (<1%). (5) From Table 4, Table 5 and Table 6, it is evident that specimens fabricated with quartz sand exhibited consistently higher peak strength values compared to those prepared with aeolian sand across all three distinct specimen types.

### 3.2. Stress-Strain Characteristics Analysis Under Different Preparation Methods

**Figure 3:**
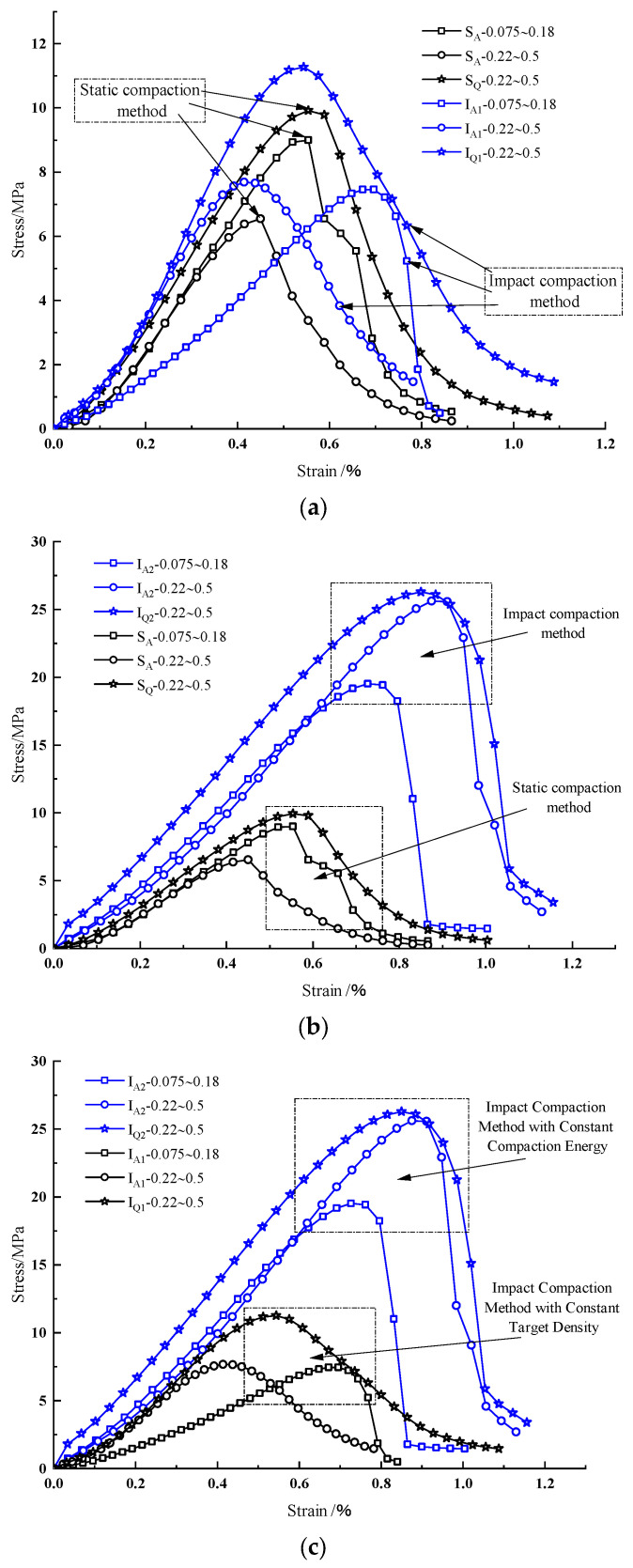
Stress-strain curves of each specimen. (**a**) Stress-strain curves of specimens prepared by impact and static compaction methods at identical densities; (**b**) Stress-strain curves of specimens prepared by impact and static compaction methods under identical compaction energy; (**c**) Stress-strain curves of specimens prepared by two impact compaction methods.

Figure 3 illustrates the stress-strain curves of tested specimens, revealing the following observations: (1) Post-Peak Brittleness: Specimens prepared via impact compaction with fine-grained aeolian sand (0.075–0.18 mm) exhibited significantly higher post-peak brittleness compared to those using coarse-grained aeolian sand (0.22–0.5 mm) or quartz sand (Figure 3a). Notably, under equivalent compaction energy, specimens displayed greater brittleness than those produced by static compaction (Figure 3b). (2) Particle Size Effects: Static-compacted specimens demonstrated increased plasticity and gradual yielding as aeolian sand particle size increased. Coarse-grained aeolian sand and quartz sand specimens exhibited prolonged failure processes with pronounced plastic deformation. (3) Compaction Energy Influence: Impact-compacted fine-grained aeolian sand specimens showed markedly higher brittleness than their coarse-grained and quartz sand counterparts (Figure 3c). Specimens compacted with identical energy exhibited enhanced brittleness, particularly for coarse-grained aeolian sand and quartz sand.

Deformation and failure processes for specimens prepared via the static compaction and impact compaction methods exhibited gradual progression, with stress-strain curves delineating four distinct phases: initial pore/microcrack compaction, elastic deformation with stable microcrack evolution, plastic deformation, and accelerated crack propagation leading to failure. (1) Initial Compaction Phase: Stress-strain curves for both methods displayed concave-downward trends due to progressive closure of inherent pores and microcracks under loading. Specimens prepared with equivalent compaction energy lacked this concavity, mirroring the behavior of intact, low-porosity natural rocks (analogous to impact-compacted specimens) compared to fractured analogs (resembling static-compacted specimens). (2) Elastic Deformation Phase: Specimens compacted with equivalent energy demonstrated prolonged elastic phases compared to static-compacted counterparts, indicating enhanced load-bearing capacity. (3) Plastic Deformation Phase: The upper stress limit of this phase corresponded to peak strength, with the transition from elastic to plastic behavior defining the yield point (stress at yield strength). (4) Post-Failure Phase: Load-bearing capacity declined rapidly with increasing deformation but remained non-zero, reflecting residual strength characteristics of fractured rock analogs.

### 3.3. Failure Mode Analysis Under Different Preparation Methods

**Figure 4:**
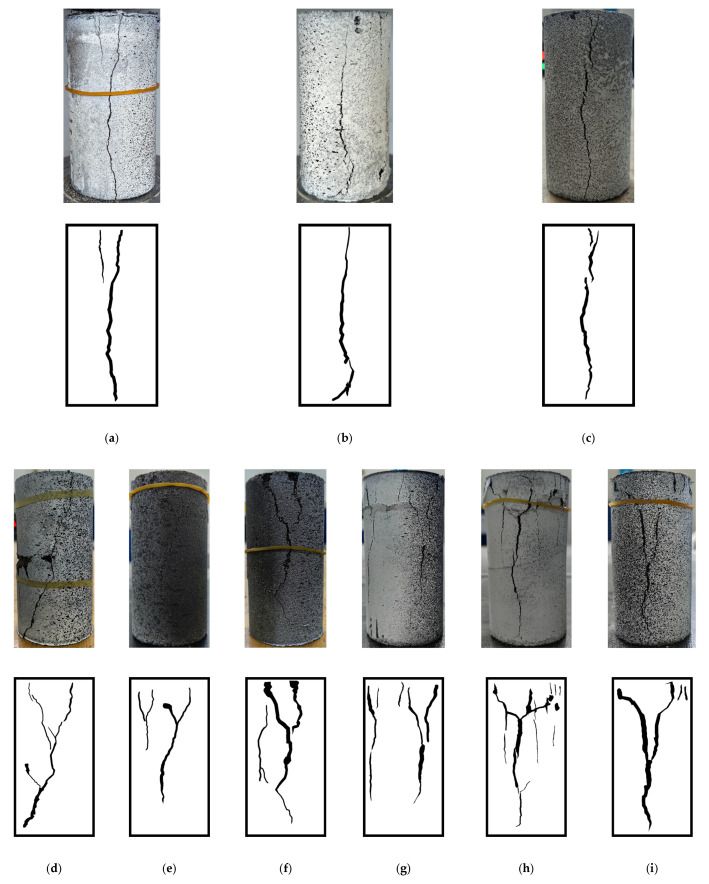
Picture of final rupture of specimens. (**a**) S_A_-0.075~0.18; (**b**) S_A_-0.22~0.5; (**c**) S_Q_-0.22~0.5; (**d**) I_A1_-0.075~0.18; (**e**) I_A1_-0.22~0.5; (**f**) I_Q1_-0.22~0.5; (**g**) I_A2_-0.075~0.18; (**h**) I_A2_-0.22~0.5; (**i**) I_Q2_-0.22~0.5.

Rock fracture involves complex processes of crack initiation, propagation, and coalescence, necessitating systematic investigation into damage evolution mechanisms. This section focuses on analyzing differences in failure morphologies between specimens prepared via distinct methodologies [34,35]. Figure 4 displays the post-test failure patterns. Specimens fabricated by the static compaction method universally failed through tensile splitting, characterized by a single vertical crack penetrating the entire specimen and aligned approximately parallel to the loading axis. This resulted in two symmetrical fracture surfaces (Figure 4a–c), attributable to the anisotropic structure formed by particle alignment during static compaction. In contrast, impact-compacted specimens (Figure 4d–i) exhibited markedly distinct failure characteristics: (1) Shear-dominated failure modes, including symmetrical Y-shaped shear bands. (2) Prominent secondary crack networks, with primary fractures generating abundant branching microcracks, leading to multiple irregular fracture surfaces. (3) Partial non-penetrating vertical cracks accompanied by pervasive surface spalling. Compared to static-compacted specimens, impact-compacted variants demonstrated larger localized crack apertures and more severe damage, attributed to altered particle interlocking states induced by cyclic impact forces during preparation.

## 4. DIC-Based Analysis of Strain Field Evolution and Crack Propagation Mechanisms Under Different Preparation Methods

### 4.1. Analysis of Crack Evolution Under Different Preparation Methods

**Figure 5:**
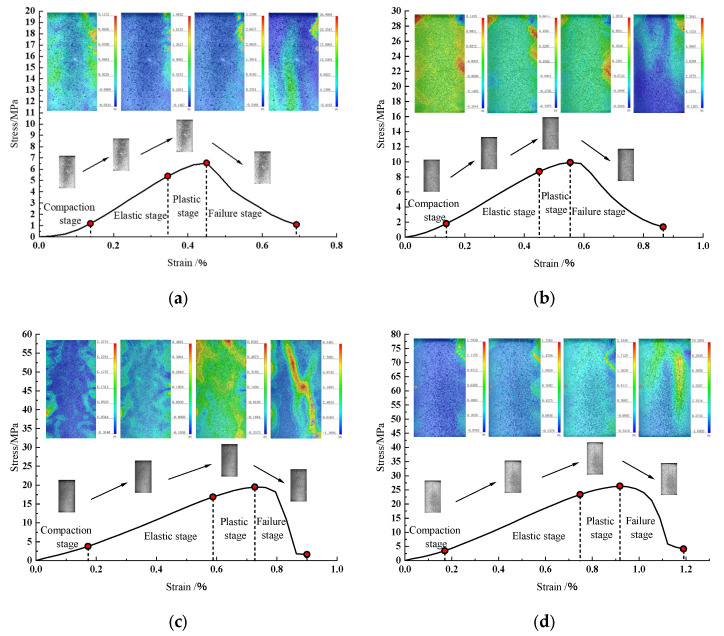
Evolution of principal strain field in four different specimens. (**a**) S_A_-0.22~0.5; (**b**) S_Q_-0.22~0.5; (**c**) I_A2_-0.075~0.18; (**d**) I_Q2_-0.22~0.5.

DIC full-field strain maps enable intuitive analysis of local crack initiation, propagation, and coalescence in specimens [36]. As established in Section 3.3, specimens prepared via identical methods exhibit significant similarity in failure modes. Therefore, this section systematically compares the evolution of maximum principal strain fields on specimen surfaces throughout the loading process by selecting two representative specimens from each compaction method (static and impact), including fine-grained aeolian sand (0.075–0.18 mm), coarse-grained aeolian sand (0.22–0.5 mm), and quartz sand (0.22–0.5 mm). The strain evolution characteristics during the compaction, elastic deformation, plastic deformation, and post-failure stages are illustrated in Figure 5.

For static-compacted specimens (Figure 5a,b), significant deformation occurred during the initial compaction phase under axial compression, with highly heterogeneous local strain distributions and pronounced spatial strain field heterogeneity, closely linked to the oriented particle arrangement formed during static compaction. In contrast, impact-compacted specimens (Figure 5c,d) exhibited relatively uniform strain distributions in the initial phase due to reduced internal porosity and homogeneous particle packing.

For aeolian sand specimens of different particle sizes (0.075–0.18 mm and 0.22–0.5 mm), static-compacted specimens (Figure 5a) retained high-strain zones from the compaction phase into the failure phase, with strain magnitudes progressively increasing. Impact-compacted specimens (Figure 5c), however, showed no such persistence beyond the plastic phase. During the elastic phase, strain heterogeneity increased in impact-compacted specimens, marked by multi-core strain concentration zones indicating uneven interparticle interlocking forces. Static-compacted specimens exhibited reduced strain heterogeneity, trending toward uniformity, though no large deformations indicative of macroscopic cracks were observed.

In the post-peak phase, static-compacted specimens (Figure 5a,b) displayed abrupt strain field transitions to bipolar patterns, with primary cracks rapidly propagating along pre-existing weak planes to form through-going tensile fractures. Impact-compacted specimens (Figure 5c,d) developed hierarchical fractal strain field structures, characterized by coordinated evolution of shear bands and secondary tensile cracks. Both methods shared rapid crack development and localized high-strain zones, manifesting as visible surface fractures.

### 4.2. Discrimination of Crack Types Under Different Preparation Methods

**Figure 6:**
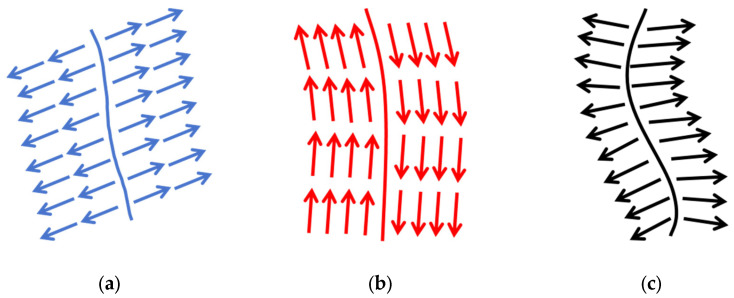
Mechanical models of different types of cracks. (**a**) Tensile crack; (**b**) Shear crack; (**c**) Mixed tensile-shear crack.

The relative displacement evolution characteristics at characteristic points (artificially defined as a, b, c…) on opposing crack faces enable quantitative discrimination of crack types. Kinematic analysis of relative displacement vectors allows refined classification: Tensile cracks exhibit separation-opening trends, commonly observed in surface spalling under low confining pressure or single tensile fractures during hydraulic fracturing initiation. Shear cracks display sliding-offset trends, typically developing in fault zones or forming conjugate shear networks under impact loading. Tension-shear composite cracks combine both displacement modes, frequently occurring in the progressive failure of jointed rock masses or multi-mode crack interactions under blasting loads. As illustrated in Figure 6 (Reference [37]), this section analyzes the crack types corresponding to failure modes of impact- and static-compacted specimens from Section 4.1 [38].

**Figure 7:**
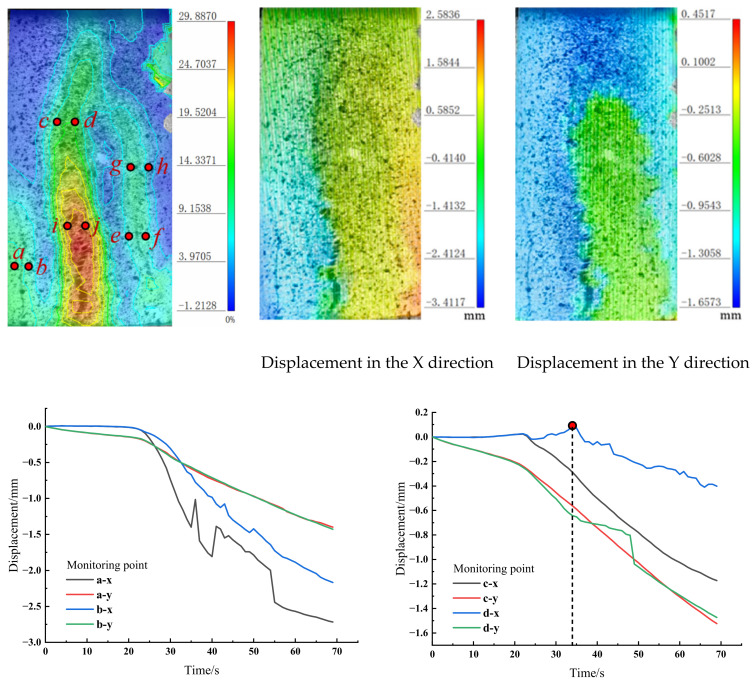

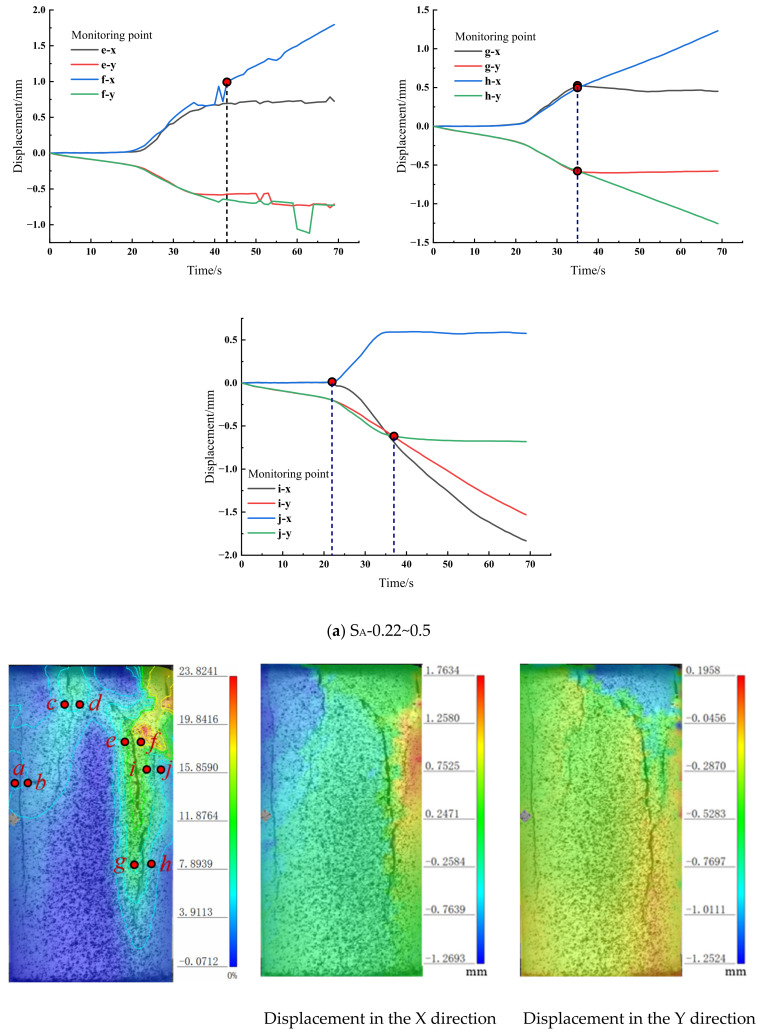

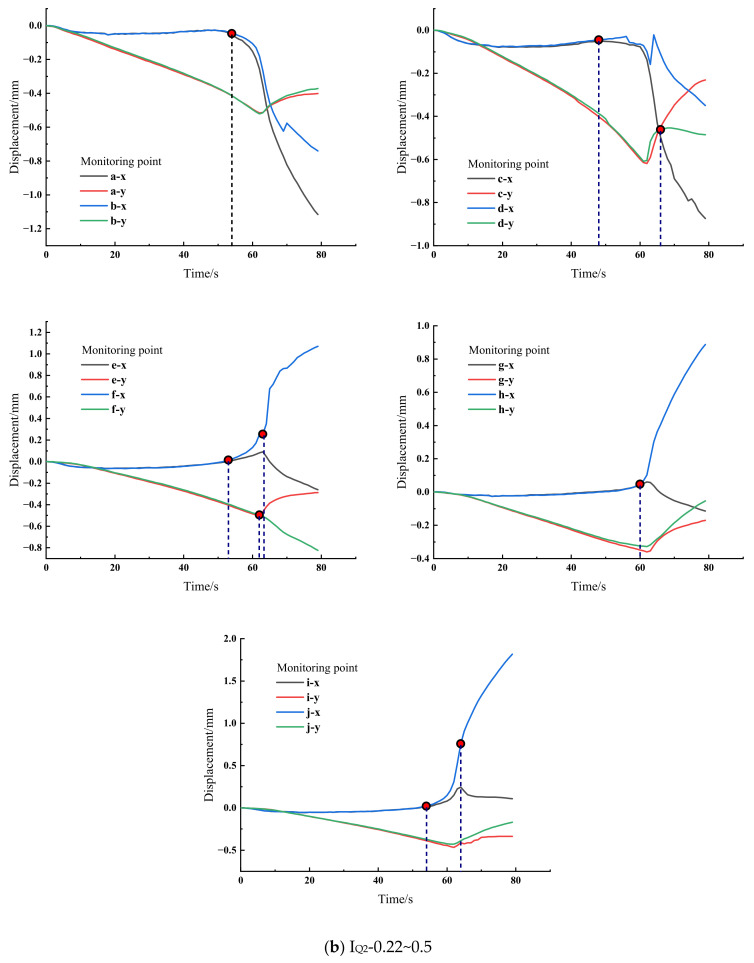
Displacement curves of points on two sides of the fracture.

DIC observations of static-compacted coarse-grained aeolian sand specimens (0.22–0.5 mm) revealed an upward crack propagation process, forming a primary vertical fracture approximately parallel to the loading direction (maximum strain concentration). For specimen C_A_-0.22–0.5, characteristic point locations, relative displacement curves, and X/Y-direction displacements are shown in Figure 7a. At points a-b, horizontal displacements on both crack faces decreased asymmetrically (a > b), indicating tensile opening due to a tensile crack initiating beneath the central fissure under boundary constraints, driving leftward displacement of the adjacent rock mass. At i-j, opposing horizontal displacements (i: leftward; j: rightward) generated significant tensile opening (i displacement ≫ j). At c-d, rapid lower crack propagation caused divergent horizontal displacements: c (left side) decreased monotonically, while d (right side) initially increased before declining. By 34 s, right-side crack development triggered a slight leftward specimen shift.

At e-f and g-h, symmetric horizontal displacement increases occurred. Post-43 s, displacement at e stabilized while f continued moving rightward, progressively widening the crack aperture. At g-h, displacement increased post-35 s due to synergistic crack propagation and central fissure effects. Vertical displacements remained synchronized at a-b, c-d, and e-f, confirming the absence of shear slip. At i-j, vertical displacements lagged horizontal movements by 15 s, reflecting slow normal-direction separation from rough fracture surfaces. Shear displacement emerged at g-h, coinciding with horizontal motion. Crack classification: Crack-a-b: Tension-dominated failure. Crack i-j → c-d: Transition from tension-shear composite to pure tensile failure. Crack e-f → g-h: Evolution from tension-dominated to tension-shear composite failure.

DIC observations of impact-compacted quartz sand specimens (S_Q2_-0.22–0.5) revealed downward shear-dominated crack propagation, generating multiple fracture surfaces with the highest principal strain localized at the upper-right corner. As shown in Figure 7b, four cracks developed in total—the highest among all specimens. Crack initiation began at c-d (48 s), where horizontal displacements decreased asymmetrically (c > d), inducing tensile opening. Subsequently, cracks formed at a-b, e-f, and i-j; e-f and i-j exhibited identical horizontal displacement growth rates along the *x*-axis, transitioning from crack nucleation to rapid propagation within 10 s. e-f: Opposing horizontal displacements (positive/negative) generated tensile opening, triggering rightward specimen shift. By 62 s, accelerated propagation caused point j to move rightward faster than i. i-j: Symmetric horizontal displacement increases maintained tensile opening. g-h: Emerged at 60 s, propagating from e-f within 7 s under sustained tensile opening. At c-d, vertical displacement divergence (shear offset) initiated at 66 s (18 s post-tensile opening), resulting in tension-shear composite failure. Points a-b, g-h, and i-j showed synchronized vertical displacements without shear slip, while e-f exhibited shear dislocation (vertical displacement lagging horizontal motion by 9 s, ≈1/3 of horizontal magnitude). Crack classification: Crack a-b: Tensile failure. Crack c-d: Tension-shear composite failure. Crack e-f → g-h: Transition from tension-shear to tension-dominated failure. Crack i-j: Tensile failure.

### 4.3. Strain Evolution Analysis at Characteristic Points Under Different Preparation Methods

**Figure 8:**
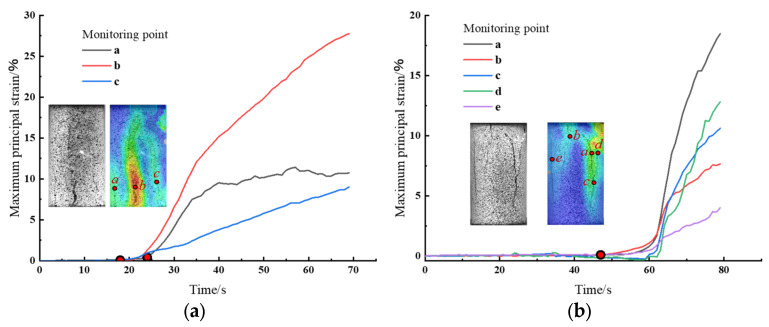
Strain curve on fracture propagation path. (**a**) S_A_-0.22~0.5; (**b**) I_Q2_-0.22~0.5.

Strain evolution curves at characteristic points quantitatively determined crack initiation timing and propagation sequences. Given overlapping initiation times across preparation methods, representative specimens with extended temporal spans from Section 4.2 were selected for analysis. Strain evolution curves for primary and secondary crack paths are illustrated in Figure 8.

Static compaction specimen (Figure 8a): The self-similar propagation phase lasted 18 s (shortest among all specimens), with minimal slow-growth stages at points b and c. Crack a: An inflection point emerged at 24 s (axial load: 2 MPa, 30% of initial yield strength), triggering localized deformation and specimen rupture. Crack b: Exhibited identical strain growth timing to Crack a but faster propagation, forming the largest central fracture. Crack c: Strain accumulation initiated at 18 s but progressed more slowly than a/b, resulting in a smaller final aperture.

Impact compaction specimen (Figure 8b): self-similar propagation phase, extended duration compared to static-compacted specimens. Crack Initiation Sequence: Cracks a, b, and e: Strain first increased at b, followed by synchronous inflection points at a/e (65 s). Cracks c (a-extension) and d: Rapid propagation post-62 s, with d surpassing c in final aperture. Fracture Hierarchy: Maximum apertures at a/c, followed by d, b, and e in descending order.

## 5. Microstructural Properties

### 5.1. Microstructural Analysis of Specimens Prepared by Different Methods

**Figure 9:**
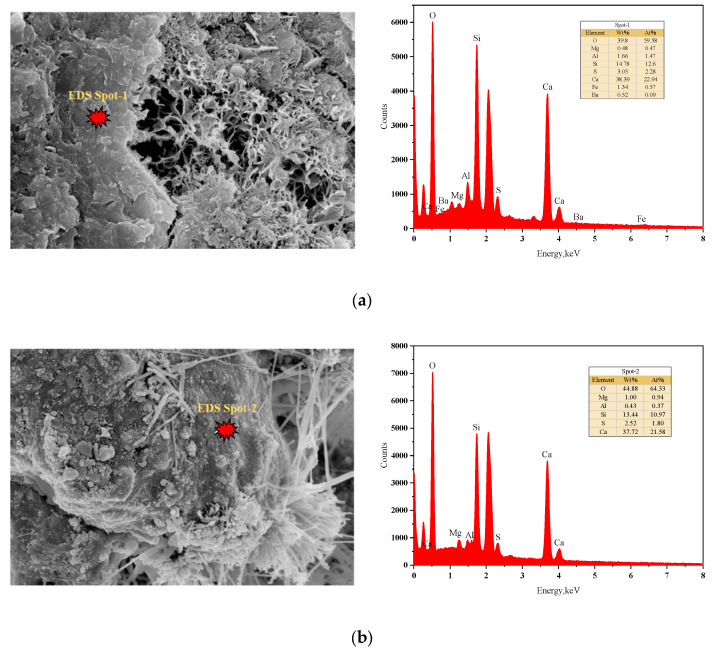
EDS spectrum of the selected samples. (**a**) EDS spectrum of the S_A_-0.075~0.18 specimen; (**b**) EDS spectrum of the I_Q_-0.22~0.5 specimen.

Rocks are naturally occurring aggregates of minerals or glass with stable morphology. Their mechanical properties are influenced by mineral composition and microstructure. Samples from the aforementioned rock-like specimens were analyzed using Energy Dispersive Spectroscopy (EDS) to determine elemental compositions and Scanning Electron Microscopy (SEM) to observe mineral morphology. Figure 9a,b present EDS results for specimens fabricated from two distinct materials, revealing elemental distributions and hydration products through diffraction peaks [39].

**Figure 10:**
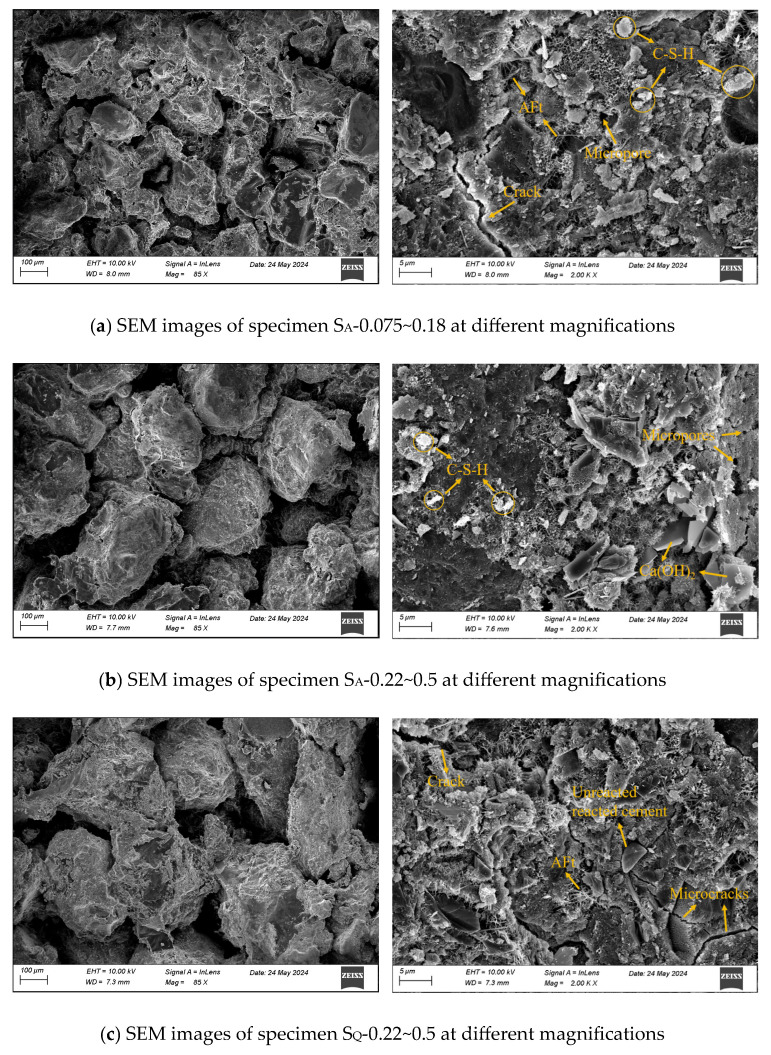

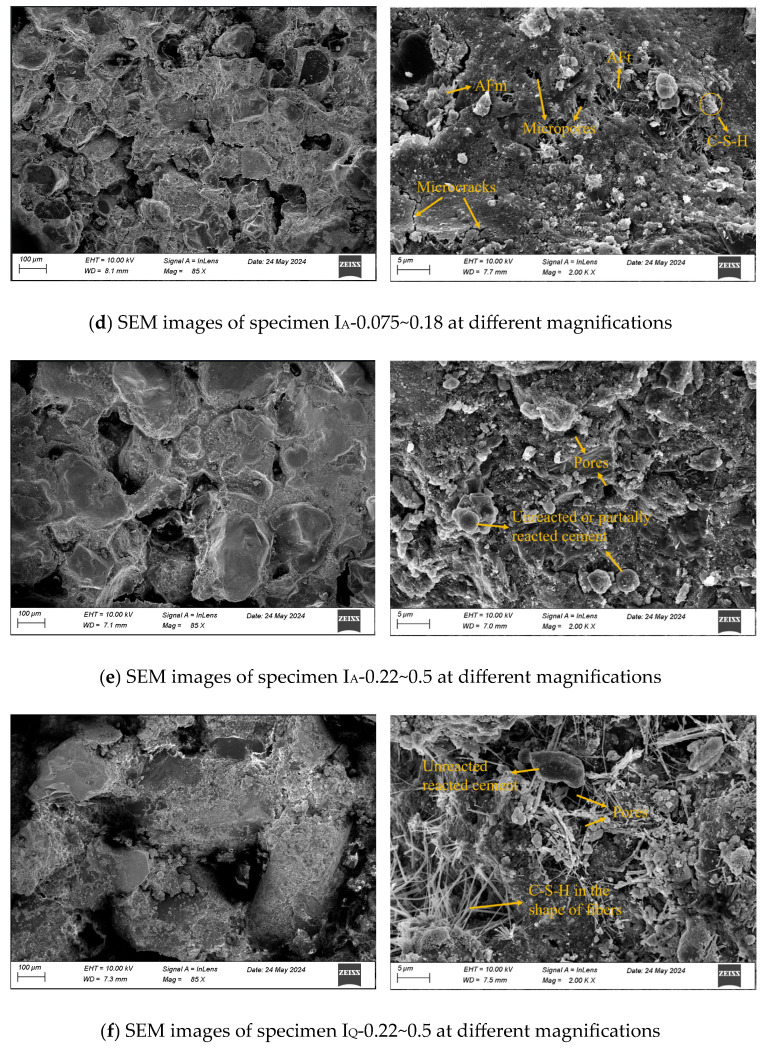
SEM images of the selected samples. (**a**) S_A_-0.075~0.18, (**b**) S_A_-0.22~0.5, (**c**) S_Q_-0.22~0.5, (**d**) I_A_-0.075~0.18, (**e**) I_A_-0.22~0.5, (**f**) I_Q_-0.22~0.5.

Key findings from SEM observations at 100 μm resolution (Figure 10) include: Porosity and Cementation: Specimens with identical aggregates and particle sizes prepared via the impact compaction method exhibited fewer pores and microvoids, along with enhanced inter-aggregate cementation, compared to those prepared by the static compaction method. Under static compaction, increasing aeolian sand particle size reduced inter-aggregate cementation, whereas larger particles in impact-compacted specimens improved cementation and reduced microvoids. For specimens with the same particle size but different materials, static-compacted quartz sand specimens demonstrated superior particle connectivity compared to aeolian sand counterparts, which exhibited looser packing. This difference diminished in impact-compacted specimens. Microscale Analysis (5 μm): Aeolian sand-based specimens exhibited layered calcium hydroxide (CH), needle-like ettringite (AFt), and flocculent calcium silicate hydrate (C-S-H gel). Static-compacted specimens (Figure 10a,b) displayed loose structures with visible microcracks and pores, forming mechanical weak points. Impact-compacted specimens (Figure 10d,e) showed denser material interfaces and enhanced structural integrity.

### 5.2. Correlation Between Synthetic Specimens and Natural Rock Types

**Figure 11:**
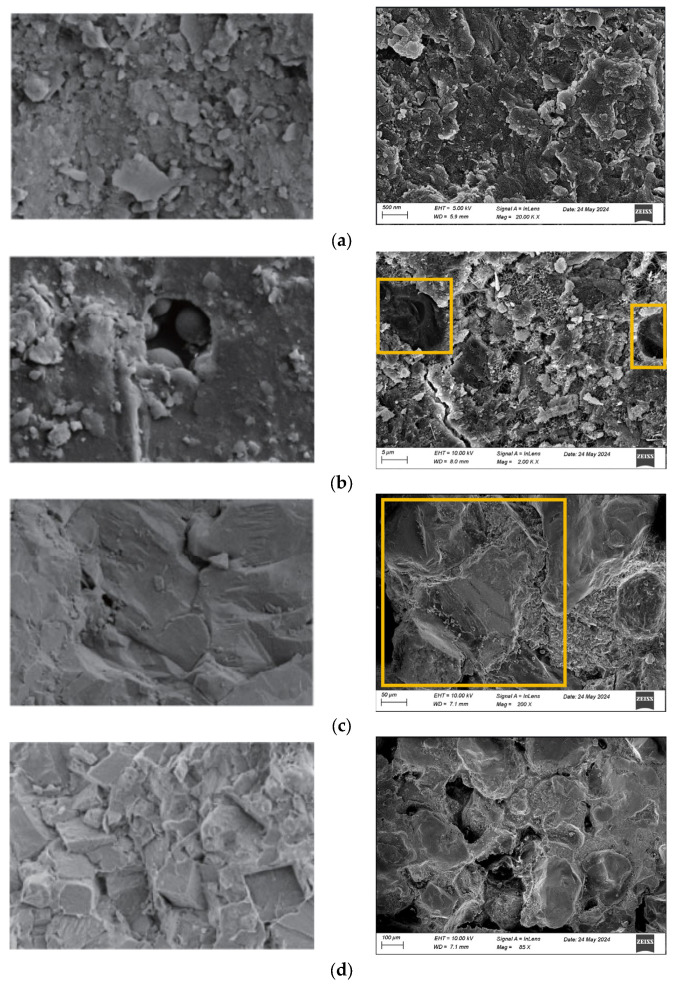
Comparative SEM images of microstructural features in different rock textures. (**a**) Root-like structure; (**b**) Small dissolution cavity structure; (**c**) Step-like structure; (**d**) Hazy zone structure.

Microstructural Comparison Between Synthetic and Natural Rock Analogs To establish correlations between laboratory-fabricated rock-like specimens and natural lithologies, a comparative microstructural analysis was conducted using SEM images (Figure 11). The left panel displays natural rock structures from Reference [40], while the right panel presents synthetic analogs. Static Compaction (Fine-Grained Aeolian Sand, 0.075–0.18 mm; Figure 11a): Exhibited a root-like structure characterized by: Loose internal packing with prominent pores and fractures, Weak interparticle cementation, High compressibility, Closely resembles weakly consolidated sedimentary rocks or heavily weathered formations. Impact Compaction (Coarse-Grained Aeolian Sand, 0.22–0.5 mm; Figure 11c): Demonstrated a stepped structure featuring: dense particle arrangement with localized integrity, Blocky aggregates, and minimal porosity (pores undetectable at 50 μm resolution). Enhanced mechanical interlocking, effectively simulates intact metamorphic rocks or strongly cemented sandstones with high strength.

**Figure 12:**
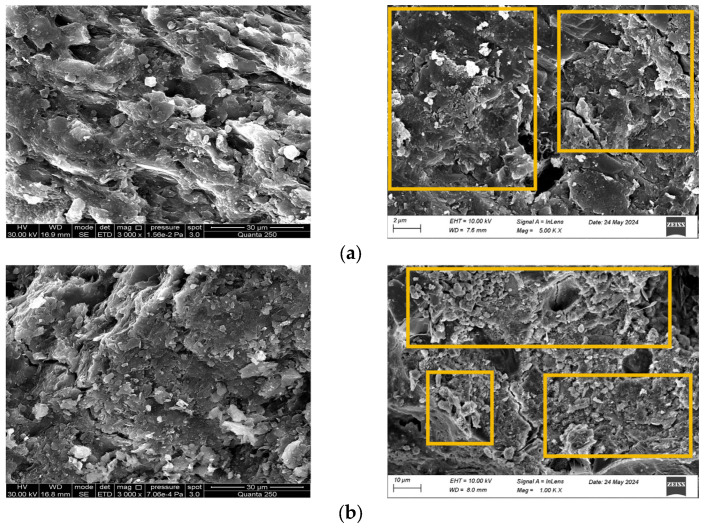
SEM comparison of different rock types. (**a**) Single-layer water-adsorbed microstructure of mudstone; (**b**) Triple-layer water-adsorbed microstructure of mudstone.

As illustrated in Figure 12, the left panel depicts natural rock structures from Reference [41], while the right panel displays laboratory-fabricated analogs. Static Compaction Method: Recommended for simulating low-strength, high-porosity rocks such as schist, shale, three-layer water-adsorbed mudstone, and fractured brecciated rocks. Fine-grained aeolian sand specimens (0.075–0.18 mm; Figure 12b) exhibit a microstructure analogous to three-layer water-adsorbed mudstone, characterized by: Loosely packed clay mineral aggregates Interconnected macropores and fractures forming distinct fracture networks Structural degradation from an initially cohesive state to a weakened configuration with pervasive cross-cutting cracks (Figure 12a → Figure 12b). Impact Compaction Method: Suitable for modeling high-strength lithologies such as single-layer water-adsorbed mudstone, granite, and basalt. Fine-grained specimens (0.075–0.18 mm; Figure 12a) demonstrate microstructural similarity to single-layer water-adsorbed mudstone, featuring: Dense clusters of clay minerals with flocculent morphology Limited interparticle porosity Enhanced mechanical interlocking compared to static-compacted counterparts.

## 6. Discussion

This study strictly adhered to standardized protocols, with three replicate specimens prepared and tested for each sample type. The mechanical properties of aeolian sand and quartz sand specimens with varying particle sizes (0.075–0.18 mm and 0.22–0.5 mm) under static compaction and impact compaction were compared, focusing on elastic modulus, peak strength, stress-strain behavior, failure modes, surface deformation fields, crack propagation paths, and strain evolution at characteristic points, thereby advancing the understanding of the relationships among particle size, preparation methodology, and failure characteristics. Synchronized observations of DIC full-field strain, crack propagation paths, and SEM microstructures, combined with comparisons to natural rock structures and lithological morphologies, rigorously validated the applicability of both methods. Recommendations were proposed: the static compaction method is suitable for simulating low-strength rocks with high porosity and microvoids, while the impact compaction method can model higher-strength rocks, providing novel insights for refining rock mechanics testing standards. Limitations include the limited number of specimens and low applicability to additional materials or particle sizes. Future studies should systematically investigate four particle size ranges (below 0.075 mm, 0.075–0.18 mm, 0.18–0.22 mm, and 0.22–0.5 mm) and diverse rock-like aggregates, extend testing to triaxial compression to analyze confining pressure effects, and prioritize large-scale specimen preparation using both compaction methods—a key challenge for further research.

## 7. Conclusions

This study investigated the effects of the impact compaction and static compaction methods on the mechanical properties and crack evolution of rock-like materials with varying particle sizes. Uniaxial compression tests integrated with digital image correlation (DIC) technology were conducted on specimens of two aeolian sand gradations (0.075–0.18 mm and 0.22–0.5 mm) and one quartz sand gradation (0.22–0.5 mm) prepared under both compaction methods. The research systematically evaluated elastic modulus, peak strength, stress-strain behavior, failure modes, surface deformation fields, crack propagation paths, and strain evolution at characteristic points. Additionally, the microstructures of synthetic specimens were analyzed and compared with natural rock analogs. Key conclusions were as follows:

1. For identical materials and particle sizes at a density of 1.82 g/cm^3^, specimens of fine-grained aeolian sand (0.075–0.18 mm) prepared by the static compaction method exhibited higher elastic modulus, initial yield strength, and peak strength compared to those fabricated by the impact compaction method. Conversely, for coarse-grained aeolian sand (0.22–0.5 mm) and quartz sand (0.22–0.5 mm) specimens, the impact compaction method resulted in higher elastic modulus, initial yield strength, and peak strength than the static compaction method. Under the static compaction method at 1.82 g/cm^3^, fine-grained aeolian sand specimens demonstrated superior elastic modulus, initial yield strength, and peak strength compared to coarse-grained aeolian sand specimens. However, under the impact compaction method, this trend reversed, with coarse-grained aeolian sand specimens achieving higher mechanical properties than their fine-grained counterparts. Under identical compaction energy (254.8 J), quartz sand specimens exhibited the highest elastic modulus and peak strength, followed by coarse-grained aeolian sand specimens, while fine-grained aeolian sand specimens displayed the lowest values in both parameters.
2. Specimens prepared by the static compaction method at a density of 1.82 g/cm^3^ (fine-grained aeolian sand, coarse-grained aeolian sand, and quartz sand) predominantly failed through tensile splitting, forming a single vertical crack approximately parallel to the loading direction that penetrated the entire specimen. In contrast, specimens prepared by the impact compaction method at a density of 1.82 g/cm^3^ and compaction energy of 254.8 J (fine-grained aeolian sand, coarse-grained aeolian sand, and quartz sand) exhibited shear-dominated failure modes, characterized by symmetrical Y-shaped shear bands or multiple fracture surfaces with higher crack density and larger localized crack apertures. DIC analysis further discriminated crack types: static-compacted specimens primarily underwent tension-controlled failure, while impact-compacted specimens displayed tension-shear composite failure mechanisms.
3. Comparative analysis with natural rock structures and lithological morphology systematically validated the applicability of both preparation methods. Based on these findings, we proposed the following recommendations: Static compaction is recommended for simulating low-strength rocks with high porosity and microvoids, such as schist, shale, three-layer water-adsorbed mudstone, and fractured brecciated rocks. Impact compaction is suitable for modeling high-strength lithologies, including single-layer water-adsorbed mudstone, granite, and basalt.

## Figures and Tables

**Table 1:** Basic physical properties of aeolian sand.

Parameter	Porosity/%	Compact Bulk Density/(kg·m^−3^)	Loose Bulk Density/(kg·m^−3^)	Apparent Density/(kg·m^−3^)	Clay Content/%	Fineness Modulus
Measured Value	44.87	1469	1307	2665	3.6	0.252

**Table 2:** Main chemical composition of quartz sand.

Component	SiO_2_	Al_2_O_3_	Fe_2_O_3_	CaO	K_2_O	Na_2_O	Other
Content/%	99.86	0.05	0.011	0.013	0.017	0.014	0.0293

**Table 3:** Specimen parameters under impact and static compaction methods at identical density.

Specimen	Specimen Preparation Method	Aggregate Type	Aggregate Particle Size (mm)	Density (g/cm^3^)	Elastic Modulus (GPa)	Initial Yield Strength (MPa)	Peak Strength (MPa)
S_A_-0.075~0.18	static compaction method	aeolian sand	0.075~0.18	1.82	2.177	7.81	9.08
I_A1_-0.075~0.18	impact compaction method	aeolian sand	0.075~0.18	1.82	1.44	6.23	7.47
S_A_-0.22~0.5	static compaction method	aeolian sand	0.22~0.5	1.82	1.963	5.39	6.57
I_A1_-0.22~0.5	impact compaction method	aeolian sand	0.22~0.5	1.82	2.473	6.43	7.7
S_Q_-0.22~0.5	static compaction method	quartz sand	0.22~0.5	1.82	2.208	8.71	9.92
I_Q1_-0.22~0.5	impact compaction method	quartz sand	0.22~0.5	1.82	2.866	9.66	11.28

**Table 4:** Static-compacted specimen parameters with varying aggregate gradations at identical density.

Specimen	Specimen Preparation Method	Aggregate Type	Aggregate Particle Size (mm)	Density (g/cm^3^)	Elastic Modulus (GPa)	Initial Yield Strength (MPa)	Peak Strength (MPa)
S_A_-0.075~0.18	static compaction method	aeolian sand	0.075~0.18	1.82	2.177	7.81	9.08
S_A_-0.22~0.5	static compaction method	aeolian sand	0.22~0.5	1.82	1.963	5.39	6.57
S_A_-0.22~0.5	static compaction method	aeolian sand	0.22~0.5	1.82	1.963	5.39	6.57
S_Q_-0.22~0.5	static compaction method	quartz sand	0.22~0.5	1.82	2.208	8.71	9.92

**Table 5:** Impact-compacted specimen parameters with varying aggregate gradations at identical density.

Specimen	Specimen Preparation Method	Aggregate Type	Aggregate Particle Size (mm)	Density (g/cm^3^)	Elastic Modulus (GPa)	Initial Yield Strength (MPa)	Peak Strength (MPa)
I_A1_-0.075~0.18	impact compaction method	aeolian sand	0.075~0.18	1.82	1.44	6.23	7.47
I_A1_-0.22~0.5	impact compaction method	aeolian sand	0.22~0.5	1.82	2.473	6.43	7.7
I_A1_-0.22~0.5	impact compaction method	aeolian sand	0.22~0.5	1.82	2.473	6.43	7.7
I_Q1_-0.22~0.5	impact compaction method	quartz sand	0.22~0.5	1.82	2.866	9.66	11.28

**Table 6:** Impact-compacted specimen parameters with varying aggregate gradations at identical compaction energy.

Specimen	Specimen Preparation Method	Aggregate Type	Aggregate Particle Size (mm)	Compaction Energy (J)	Elastic Modulus (GPa)	Initial Yield Strength (MPa)	Peak Strength (MPa)
I_A2_-0.075~0.18	impact compaction method	aeolian sand	0.075~0.18	254.8	3.157	16.87	19.54
I_A2_-0.22~0.5	impact compaction method	aeolian sand	0.22~0.5	254.8	3.48	23.14	25.71
I_A2_-0.22~0.5	impact compaction method	aeolian sand	0.22~0.5	254.8	3.48	23.14	25.71
I_Q2_-0.22~0.5	impact compaction method	quartz sand	0.22~0.5	254.8	3.511	23.33	26.29

## Data Availability

The original contributions presented in the study are included in the article. Further inquiries can be directed to the corresponding authors.
